# Accurate late gadolinium enhancement prediction by early T1- based quantitative synthetic mapping

**DOI:** 10.1007/s00330-017-5018-2

**Published:** 2017-08-30

**Authors:** Randy van Dijk, Dirkjan Kuijpers, Theodorus A. M. Kaandorp, Paul R. M. van Dijkman, Rozemarijn Vliegenthart, Pim van der Harst, Matthijs Oudkerk

**Affiliations:** 1 University of Groningen, University Medical Centre Groningen, Centre for Medical Imaging, Groningen, The Netherlands; 2 Department of Cardiology, University of Groningen, University Medical Centre Groningen, Groningen, The Netherlands; 3 Department of Cardiovascular Imaging HMC-Bronovo, The Hague, The Netherlands; 4 Department of Radiology, University of Groningen, University Medical Center Groningen, Groningen, The Netherlands; 5 Center for Medical Imaging, University Medical Center Groningen, Hanzeplein 1 EB 45, Groningen, The Netherlands

**Keywords:** Coronary artery disease, Magnetic resonance imaging, Adenosine, Gadolinium, Fibrosis

## Abstract

**Objectives:**

Early synthetic gadolinium enhancement (ESGE) imaging from post-contrast T1 mapping after adenosine stress-perfusion cardiac magnetic resonance (CMR) was compared to conventional late gadolinium enhancement (LGE) imaging for assessing myocardial scar.

**Methods:**

Two hundred fourteen consecutive patients suspected of myocardial ischaemia were referred for stress-perfusion CMR. Myocardial infarct volume was quantified on a per-subsegment basis in both synthetic (2–3 min post-gadolinium) and conventional (9 min post-gadolinium) images by two independent observers. Sensitivity, specificity, PPV and NPV were calculated on a per-patient and per-subsegment basis.

**Results:**

Both techniques detected 39 gadolinium enhancement areas in 23 patients. The median amount of scar was 2.0 (1.0–3.1) g in ESGE imaging and 2.2 (1.1–3.1) g in LGE imaging (*p*=0.39). Excellent correlation (r=0.997) and agreement (mean absolute difference: -0.028±0.289 ml) were found between ESGE and LGE images. Sensitivity, specificity, PPV and NPV of ESGE imaging were 96 (78.9–99.9), 99 (97.1–100.0)%, 96 (76.5–99.4) and 99.5 (96.6–99.9) in patient-based and 99 (94.5–100.0), 100 (99.9–100.0)%, 97.0 (91.3–99.0) and 100.0 (99.8–100.0) in subsegment-based analysis.

**Conclusion:**

ESGE based on post-contrast T1 mapping after adenosine stress-perfusion CMR imaging shows excellent agreement with conventional LGE imaging for assessing myocardial scar, and can substantially shorten clinical acquisition time.

***Key Points*:**

• *Synthetic gadolinium enhancement images can be used for detection of myocardial scar.*

• *Early synthetic gadolinium enhancement images can substantially shorten clinical acquisition time.*

• *ESGE has high diagnostic accuracy as compared to conventional late gadolinium enhancement.*

• *Quantification of myocardial scar with ESGE closely correlates with conventional LGE.*

• *ESGE after stress perfusion CMR avoids need for additional gadolinium administration.*

## Introduction

The clinical evaluation of chronic myocardial infarction includes the evaluation of late gadolinium enhancement (LGE) in the myocardium using contrast-enhanced cardiovascular magnetic resonance (CMR) imaging [1]. Currently, following the adenosine stress-perfusion CMR to assess myocardial viability, conventional LGE images are acquired 10–15 min after an additional bolus of gadolinium (0.05 mmol/kg) [2, 3]. Recently, a new gadolinium enhancement technique was introduced in which synthetic phase sensitive inversion recovery (IR) images were generated based on post-gadolinium T1 mapping pixel values [4–6]. This modified Look-Locker inversion recovery (MOLLI) technique creates not only fast T1 mapping of the myocardium, but also synthetic IR images that can be calculated retrospectively at any inversion time (TI) [4–6]. These synthetic IR images were originally developed to effectively register individual MOLLI images to reduce motion artefacts; however, they also contain excellent delayed enhancement contrast [7].

In a recent study, synthetic IR LGE images were compared to conventional IR LGE imaging in patients with suspected myocardial infarction, both obtained after a regular waiting time after gadolinium injection of 10–15 min. This study showed a high sensitivity (90 %) and specificity (96 %) of the synthetic IR images for the detection of LGE as compared to conventional IR LGE images [7]. Furthermore, a high and robust precision of LGE area quantification was reported using synthetic IR images over a wide range of TI [8].

We hypothesised that the timing of acquisition after intravenous gadolinium to detect myocardial scar can be significantly reduced using synthetic IR imaging. We studied whether we can advance to early synthetic gadolinium enhancement (ESGE) imaging by obtaining TI data 2 min after the first-pass perfusion imaging under adenosine stress, without an additional dose of gadolinium, with similar sensitivity and specificity to conventional LGE imaging for the assessment of myocardial scar.

## Materials and methods

### Methods

The institutional review board approved this prospective study. All patients provided written informed consent before enrolment in the study. Between August 2015 and January 2016, 214 consecutive patients suspected of myocardial ischaemia were enrolled for adenosine stress CMR, after excluding common contraindications for MR imaging, gadolinium and adenosine. Conventional LGE was considered the standard of reference in this study for the detection and quantification of myocardial scar.

### CMR imaging protocol

A 1.5 T system (Magnetom Avanto-fit; Siemens Healthcare, Erlangen, Germany) with phased-array radiofrequency coils was used in all patients. Adenosine stress-only perfusion MRI was performed according to conventional methods. A standard 40-s perfusion sequence was started during the first pass of 0.1 mmol/kg gadopentetate dimeglumine, injected at a flow rate of 5 ml/s, after 3 min of adenosine infusion. The research sequence, namely post-gadolinium T1 mapping, was performed exactly 2 min after the start of the bolus injection of gadolinium for the perfusion series. For the current study, equal position and orientation of 5 short-axis slices were used in post-gadolinium T1 mapping and the conventional LGE imaging. An additional dose of contrast material (0.05 mmol/kg) was given directly after the last T1-mapping sequence, followed by conventional LGE images 9 min after the second dose of gadolinium. Synthetic IR images were calculated automatically from the post-gadolinium T1 map.

#### Early quantitative synthetic gadolinium enhancement

After the standard adenosine stress perfusion series, an investigational modified Look-Locker inversion recovery (MOLLI)-based post-gadolinium T1-mapping sequence (WIP780B) was used to acquire a pixel-wise T1 map of the myocardium. In each patient five short-axis T1 maps were acquired equally divided over the left ventricle with a distance factor of 8 mm, during a short breath-hold (10–11 s) at end-expiration. A 4(1)3(1)2 sampling scheme of the heart was performed, including nine images in 11 heartbeats. An adiabatic inversion pulse was used to improve inversion efficiency. For the MOLLI acquisition, an initial TI of 100 ms was used with 80 ms TI increment. The images were generated using a single-shot steady-state free-precession readout. T1 maps were generated on the imaging unit by using a three-parameter signal model. The calculated T1 for each pixel (T1 map) was used to create series of quantitative synthetic IR images in short-axis view with inline respiratory motion correction. Each synthetic image series consisted of 40 images with TI increments of 25 ms, starting at 200 ms. Typical parameters were: slice thickness, 8 mm; reconstruction matrix 256 × 192; in-plane spatial resolution, 1.65 × 1.65 mm^2^; echo time 1.09 ms; bandwidth, 1,085 Hz/ pixel; flip angle, 35°; and parallel imaging acceleration factor, 2.

#### Late conventional gadolinium enhancement

Conventional LGE imaging involved a cardiac-gated single-shot gradient-echo pulse sequence with full coverage of the left ventricle, during two breath-holds at end-expiration. The sequence included nine continuous short-axis slices, in which the short-axis section position and orientation were copied from the T1-mapping acquisition. Typical parameters were: slice thickness, 8 mm; reconstruction matrix 192 × 170; in-plane spatial resolution, 1.77 × 1.77 mm^2^; TI 300 ms; echo time 1.09 ms; bandwidth, 1,085 Hz/pixel; flip angle, 65°; and parallel imaging acceleration factor, 2.

### Image analysis

**Fig. 1:**
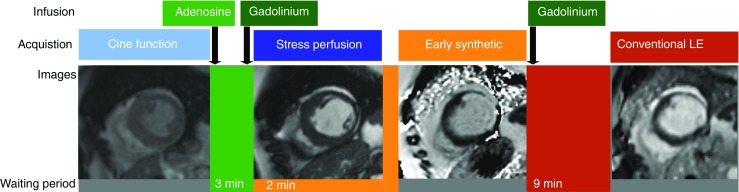
Cardiac MR imaging protocol. Cine function imaging is followed by injection of adenosine. After a waiting period of 3 min acquisition of the first-pass perfusion of gadolinium contrast is achieved. Two min after the injection of the first bolus of gadolinium, early synthetic inversion recovery (IR) images are acquired. After this, a second bolus of gadolinium is injected and after an additional waiting period of 9 min the conventional late gadolinium enhancement (LGE) IR images are acquired

ESGE and conventional LGE images were analysed by two blinded observers for agreement regarding localisation and extent of the myocardial scar. The MR protocol is shown in Fig. 1. The gadolinium enhancement areas were quantified by two radiologists with more than 10 years of experience in cardiac MR. Both readers performed quantitative analysis in a random order and were blinded to the patient data.

The images were analysed using commercially available software (MASS analytical software, Medis, Leiden, The Netherlands). Endo- and epicardial contours of the left ventricle were drawn manually. A reference point was placed outside and below the inferior wall of the left ventricle in short-axis direction. The software automatically divided the myocardium into six (basal and mid-ventricular short-axis slices) or four (apical short-axis slice) segments according to the American Heart Association’s segment model [9].

In each patient two sets (early synthetic and conventional LGE images) of five equal-positioned short-axis slices were evaluated. At both basal and mid-ventricular level two short-axis slices each were taken, and one slice at apical level, which gave a total of 28 subsegments per left ventricle.

A binary threshold algorithm that uses five standard deviations above the average signal intensity of the normal myocardium was applied to automatically delineate the area showing gadolinium enhancement [10]. Pixels above this threshold limit were considered to be part of the infarcted area. These pixels were automatically counted by the software, and the total area of these pixels was calculated and then expressed as the volume (g) of the total myocardial cross-sectional area (at a slice thickness of 8 mm) in the particular section. To evaluate the area of gadolinium enhancement, the mean signal intensity in the normal myocardium was measured by using a region of interest (ROI) drawn in an area of normal myocardium containing at least 100 pixels. Gadolinium-enhanced areas were identified on a per-short-axis slice basis and quantified regions of ESGE images (derived from post-gadolinium T1 maps) were compared with conventional LGE images.

### Statistical analysis

Continuous variables without normal distribution were reported as median with interquartile range and compared by the Mann-Whitney *U* test (unpaired data) or Wilcoxon signed rank test (paired). Differences in gadolinium enhancement areas were analysed with the paired *t* test. Sensitivity, specificity, positive predictive value (PPV) and negative predictive value (NPV) were calculated on patient- and subsegment-based analysis. The correlation between ESGE and conventional LGE was evaluated with linear regression analysis using Pearson’s correlation coefficient. Interobserver variability was assessed by calculating the interclass correlation coefficient for both ESGE and LGE. To assess the agreement and potential systematic differences between the readers and imaging techniques Bland-Altman analysis was performed indicating mean difference and 95 % limits of agreement. Statistical significance was defined as a *p*-value of <0.05. Statistical analyses were performed by using SPSS statistics 23 (IBM corporation, USA).

## Results

Twenty-three (10.7 %) patients had 39 gadolinium enhancement areas consistent with myocardial scars. The mean age of the study population with myocardial scar was 68±11 years, 17 (81 %) patients were male, 4 (19 %) patients had diabetes, and the mean body mass index (BMI) was 28±5 kg/m^2^. The maximal heart rate during adenosine infusion was significantly higher compared to the resting heart rate (*p*<.0001).

### Diagnostic accuracy

The patterns of enhancement were consistent with myocardial scar in each patient. The ESGE and conventional LGE technique detected areas of enhancement in 21 corresponding patients. The ESGE images were false-negative in one patient (one subsegment) and false-positive in one patient (three subsegments).

In the 23 patients with gadolinium enhancement 644 subsegments were evaluated. Gadolinium enhancement consistent with myocardial scar were detected in 101 of 644 subsegments in 23 patients, whereas 5,348 subsegments in 191 patients (191 × 28) were considered normal. The total number of true-negative subsegments was 5,891 (543+5,348). When comparing ESGE to conventional LGE as standard of reference for detection of myocardial scar, sensitivity, specificity, PPV and NPV were 96 % (78.9–99.9), 99 % (97.1–100.0), 95.8 (76.5–99.4) and 99.5 (96.6–99.9), respectively, for patient-level analysis and 99 % (94.5–100.0), 100 % (99.9–100.0), 97.0 (91.3–99.0), and 100.0 (99.8–100.0), respectively, for segment-based analysis.

### Correlation and agreement between ESGE and LGE

**Fig. 2:**
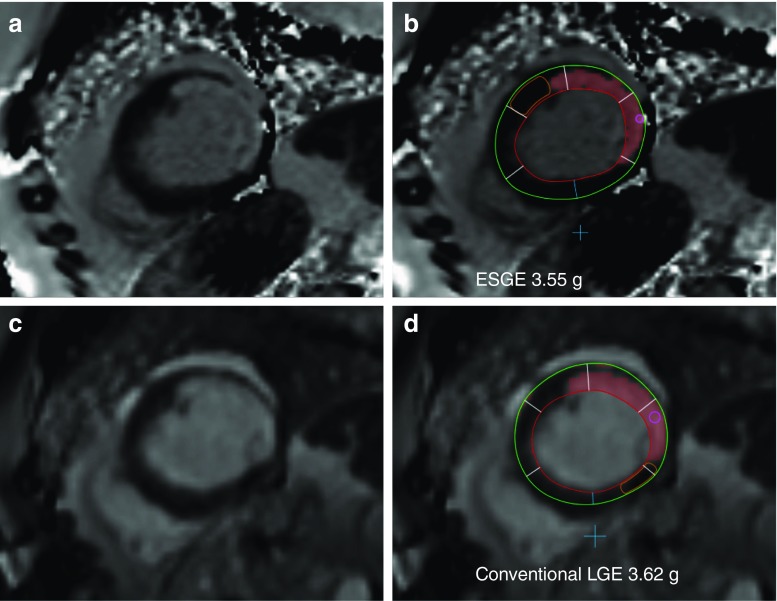
A representative mid-ventricular short-axis examination of the left ventricle of a patient with a lateral infarction of both early synthetic gadolinium enhancement (ESGE) images 2 min post-gadolinium infusion under adenosine hyperaemia (**a**) and conventional late gadolinium enhancement (LGE) images 9 min post gadolinium infusion (**c**). Quantification on a per-short-axis basis using semi-automated software is shown on both ESGE (**b**) and conventional LGE (**d**) inversion recovery (IR) images

**Fig. 3:**
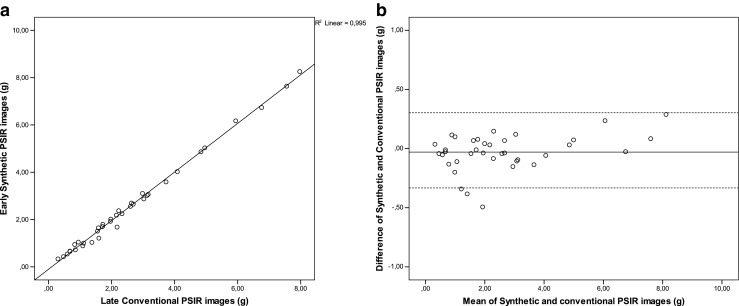
Correlation (**a**) and agreement (**b**) between early synthetic gadolinium enhancement (ESGE) and conventional late gadolinium enhancement (LGE) images

The two patients with false-negative and false-positive findings were excluded from further quantitative analysis**.** Quantitative analysis of myocardial scar was performed in 21 patients and 97 subsegments. The median amount of gadolinium enhancement was 2.0 (1.0–3.1) g in the early synthetic images and 2.2 (1.1–3.1) g in the conventional images (*p*=0.386). A representative example of gadolinium-enhancement detection by ESGE and conventional LGE is shown in Fig. 2. Excellent correlation (r=0.997) and agreement (mean difference -0.02951±0.15457 g) were found (see Fig. 3).

### Interobserver correlation and agreement

**Fig. 4:**
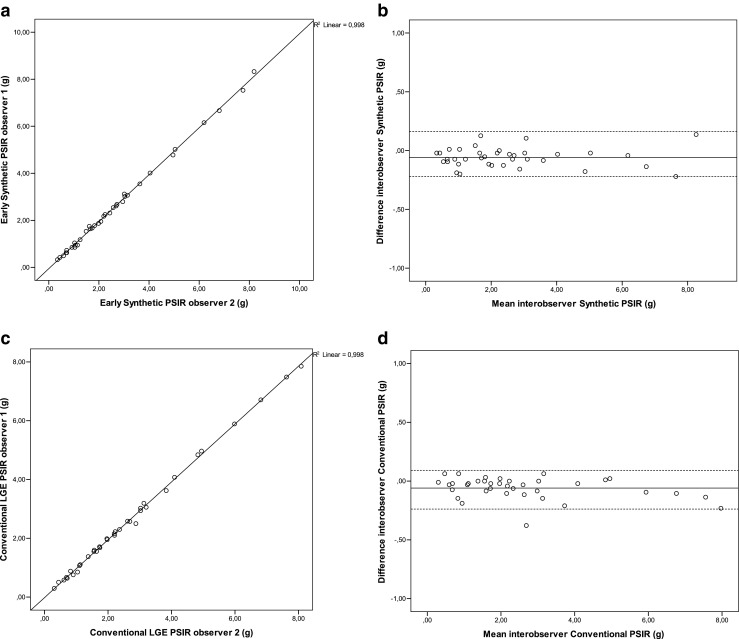
Interobserver correlation (**a**) and absolute agreement (**b**) for early synthetic gadolinium enhancement (ESGE) and interobserver correlation (**c**) and absolute agreement (**d**) for conventional late gadolinium enhancement (LGE) images

The ICC was 0.999 (0.998–1.000) at a *p*-value <.001 for both ESGE and conventional LGE with a mean difference of -0,0585±0,0826 g and -0.0596±0.09117 g , respectively. Interobserver correlation and agreement are shown in Fig. 4.

## Discussion

We demonstrated that early enhancement of myocardial scar with post-contrast T1 mapping performed directly after adenosine perfusion CMR showed excellent correlation and agreement with conventional IR LGE. Our results show high sensitivity and specificity between ESGE and conventional LGE images as assessed by semi-automated evaluation. The ESGE images can be generated directly after the stress-perfusion CMR images for the detection of myocardial scar to differentiate ischaemic perfusion defects from non-ischaemic perfusion defects.

### Reduced scan time with no additional gadolinium

The normal delay time after the stress perfusion acquisition is about 10 min, which is a considerable part of the scan time. In this study we showed that ESGE imaging generated from post-gadolinium T1 mapping may eliminate the need for conventional LGE imaging, which reduces scan time and avoids additional administration of gadolinium. The early detection of gadolinium enhancement on the T1-mapping-based images 2 min after contrast injection as compared to the conventional LGE images can be explained by several factors.

First of all, the mechanism of early enhancement relates to the different wash-in and wash-out kinetics of normal myocardium and myocardial scar. The extracellular volume fraction in areas of myocardial scar is higher than that in regions with normal myocardial tissue. The hyperaemia induced by adenosine, facilitates wash-in of gadolinium in the extracellular volume, especially in the regions of infarcted myocardium. These regions will have a higher concentration of gadolinium and subsequently greater T1 shortening [11]. Secondly, the quantitative synthetic IR technique depicts gadolinium enhancement with high sensitivity and specificity [6], which in combination with adenosine-induced hyperaemia, avoids the administration of an additional bolus of gadolinium, as required for conventional LGE imaging.

### Quantification of myocardial scar

The results in our study showed that quantification of ESGE imaging in patients with myocardial scars was feasible and reproducible. The amount of scar based on ESGE imaging was comparable to conventional single-shot PSIR LGE imaging, which was used as the standard of reference. ESGE imaging resulted in one false-negative finding. In one patient a subendocardial enhancement area (one subsegment) was not detected, probably due to the fact that this scan was performed too early after administration of gadolinium. Conventional single-shot PSIR LGE failed to view one subendocardial scar (three subsegments) in the lateral wall mainly due to the relative high density of the blood-pool contrast.

In this study we used a standard semi-automated analysis to quantify the infarct areas with commercially available software. We set a threshold of five standard deviations above the average signal intensity of the normal myocardial tissue. This threshold has been proven to be most accurate among the binary techniques in quantification of myocardial infarction [10].

Compared to the reference LGE images, ESGE images improve infarct border visualisation without oversaturation of the region of interest, especially concerning the blood pool area. The demarcation of endocardial contours in the infarcted areas in this study was more simple to perform in the ESGE images compared to the LGE images. In this study, despite the manual input to draw the inner and outer lines of the left ventricle, the interobserver correlation coefficients between synthetic and conventional images were excellent. This new quantitative T1-mapping-based enhancement technique, if used as full coverage of the left ventricle, has the potential to be used in the evaluation of myocardial viability and scar volume assessment (Table 1).

**Table 1:** Haemodynamics during rest and under adenosine hyperaemia

Haemodynamics	Rest	Adenosine	*p*-value
Systolic BP (mm Hg)	145 (128–170)	141 (125–165)	0.211
Diastolic BP (mm Hg)	81 (74–88)	81 (74–91)	0.747
Heart rate (bpm)	68 (57–85)	84 (70–101)	<.001

Values are presented as median (interquartile range)

*BP* blood pressure, *bpm* beats per minute

### Limitations

In this study we used single-shot PSIR as standard of reference for LGE. It is known that segmented 2D and 3D sequences for LGE, compared to single-shot PSIR, has a better in-plane resolution and a better definition of the infarcted area; however, in this study we used single-shot PSIR mainly because the in-plane resolution is comparable to the ESGE images.

We avoided full coverage of the left ventricle in this study protocol because we aimed to preserve a fixed short time-interval between the different gadolinium enhancement techniques. Too many T1 maps may represent different states of contrast enhancement, which could interfere with the results. The quantification of the total volume of infarct in this study was not possible because only five short-axis slices were used for analysis.

In our study a breath-hold of 10–11 s was sufficient for acquiring the synthetic PSIR images. We recognise, however, that the acquisition time of synthetic PSIR is longer compared to single-shot PSIR conventional LGE imaging. In patients with chronic obstructive pulmonary disease, heart failure or elderly patients the breath-hold instructions may be a problem.

The heart-rate increasing effects of adenosine can influence the quality of the MOLLI images. However, as shown in our results, there was a strong correlation between the ESGE and conventional LGE images without any decrease in image quality.

Because our study population consisted of patients at risk of CAD but without previous documented myocardial infarction, the detected mass of infarcted myocardium was relatively small. We showed that the correlation and agreement between ESGE and conventional LGE was excellent across the whole range of mass of infarcted myocardium. We do not expect different results in a study population with larger areas of infarcted myocardium. However, this new enhancement technique needs further validation before it can be used for full coverage of the left ventricle.

### Conclusion

The use of early quantitative synthetic IR images from post-contrast T1 mapping after adenosine perfusion CMR can substantially shorten the clinical scan time and avoid the need for an additional bolus of gadolinium. The detection of myocardial infarction as well as reproducibility and agreement of scar volume quantification in ESGE as compared to conventional single shot PSIR LGE images is excellent. Furthermore, infarct border visualisation appears to improve in ESGE images without oversaturation of the ROI.

## Abbreviations

ESGE: Early synthetic gadolinium enhancement
IR: Inversion recovery
LGE: Late gadolinium enhancement
MRI: Magnetic resonance imaging
MOLLI: Modified Look-Locker inversion recovery
NPV: Negative predictive value
PPV: Positive predictive value
TI: Inversion time
